# Ruptured Hepatic Artery Aneurysms Revealing Systemic Lupus Erythematosus

**DOI:** 10.31662/jmaj.2025-0310

**Published:** 2025-12-05

**Authors:** Saori Inoue, Mari Tatsumi, Yu Tanaka, Aya Ide

**Affiliations:** 1 Department of Internal Medicine, Meitetsu Hospital, Nagoya, Japan; 2 Department of Gastroenterology, Meitetsu Hospital, Nagoya, Japan

**Keywords:** systemic lupus erythematosus, hepatic artery aneurysm, rupture, transcatheter arterial embolization

## Abstract

Hepatic artery aneurysms are rare vascular lesions, most often caused by infection, but occasionally linked to connective tissue diseases such as systemic lupus erythematosus (SLE). We report a case of multiple hepatic artery aneurysms revealing SLE with antiphospholipid antibody syndrome. A 74-year-old woman, having previously undergone splenectomy for pancytopenia of unknown cause, was admitted for postoperative maxillary cyst infection. On day 10, she developed acute abdominal pain; computed tomography showed multiple hepatic artery aneurysms with rupture. Emergency transcatheter arterial embolization of the A5 branch achieved hemostasis. Serology was positive for antinuclear antibody, anti-double-stranded DNA antibody, lupus anticoagulant, and anti-β2-glycoprotein I antibody. She had pericardial effusion, hypocomplementemia, proteinuria, hemolytic anemia, and erythema, leading to a diagnosis of SLE with antiphospholipid antibody syndrome. Glucocorticoids and cyclophosphamide were initiated, resulting in rapid aneurysm regression and near-complete resolution at 6 months.

Review of reported SLE-associated hepatic artery aneurysm cases shows that most presented with gastrointestinal bleeding or hemobilia, whereas our patient presented with rupture and abdominal pain during hospitalization. Endovascular embolization was the preferred initial treatment, with favorable outcomes when performed promptly. The rapid regression following immunosuppression in our case supports an inflammatory vasculitic mechanism.

This case highlights the need to consider SLE in the differential diagnosis of hepatic artery aneurysms, especially when infection is excluded, and underscores the importance of rapid diagnosis, urgent hemostasis, and timely immunosuppressive therapy.

## Introduction

Hepatic artery aneurysms are most commonly caused by infection but are occasionally associated with connective tissue diseases such as systemic lupus erythematosus (SLE) ^(1), (2)^. We report a case of multiple hepatic artery aneurysms that led to the diagnosis of SLE, accompanied by a literature review.

## Case Report

A 74-year-old woman had undergone splenectomy 3 years earlier for unexplained pancytopenia and splenomegaly, at which time antinuclear antibody positivity was noted. She also had a history of chronic maxillary sinusitis (bilateral radical surgery in her teens). She presented with low-grade fever and headache, and was admitted on day 1 with a diagnosis of infection of a postoperative left maxillary cyst. On day 2, incision and drainage of the cyst were performed. Blood cultures were negative, and echocardiography revealed pericardial effusion without vegetations.

**Figure 1.**
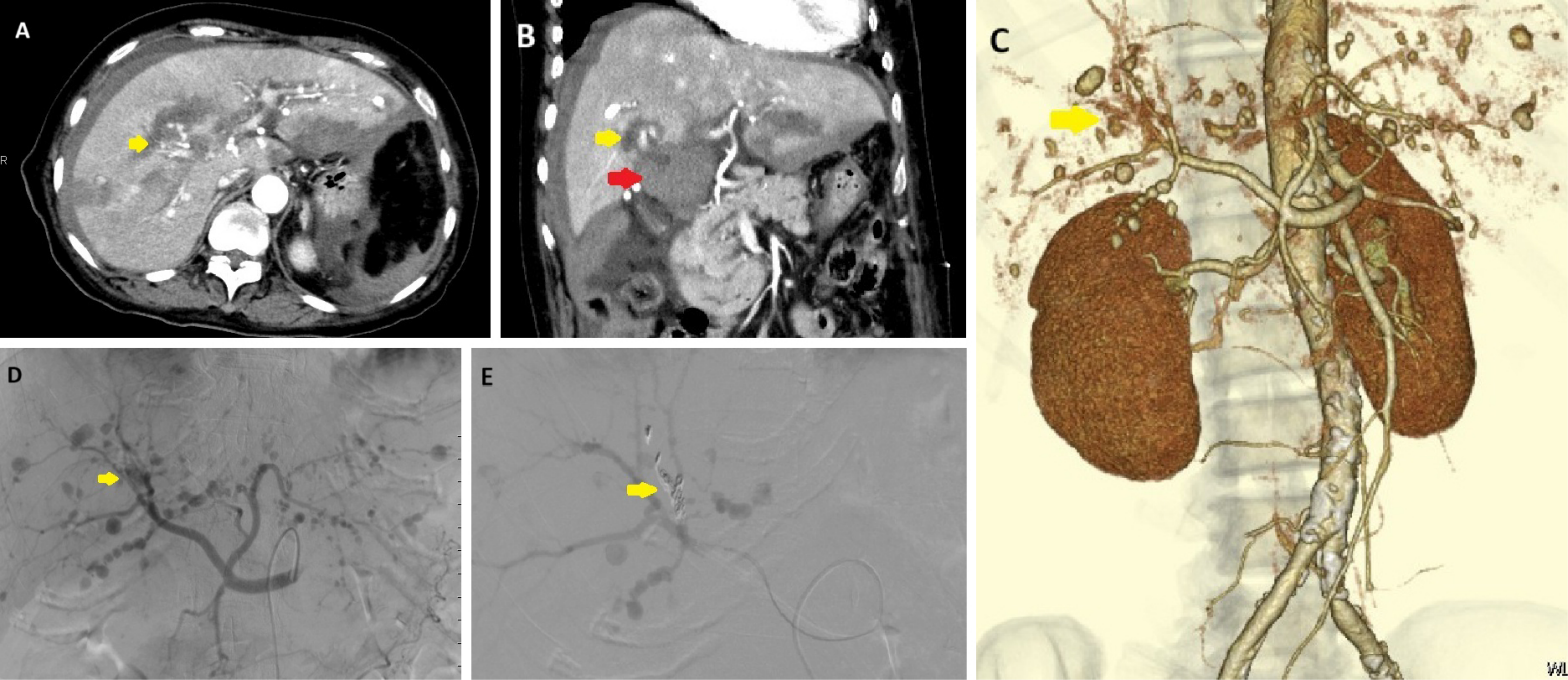
Contrast-enhanced CT and angiography at symptom onset. Contrast-enhanced CT obtained immediately after the onset of abdominal pain shows multiple hepatic artery aneurysms. (A) Axial arterial-phase image and (B) coronal arterial-phase image demonstrate several aneurysms. Intraperitoneal hemorrhage originating from the A5 branch is suspected (yellow arrow), and a hematoma is present in the hepatic hilum (red arrow). No arterial aneurysms were identified in locations other than the liver. (C) Three-dimensional reconstructed CT highlights the extravasation at the A5 branch (yellow arrow). (D) Angiography confirms contrast extravasation from the same site (yellow arrow). (E) Coil embolization was performed at the A5 branch (yellow arrow), clearly showing coil deployment at the bleeding site. CT: computed tomography.

On day 10, she developed abdominal pain. Contrast-enhanced computed tomography (CT) demonstrated multiple hepatic artery aneurysms with contrast extravasation around the perihilar region, suggesting intraperitoneal hemorrhage (Figure 1A-C). Emergency transcatheter arterial embolization (TAE) of the segment 5 branch achieved hemostasis with coil placement (Figure 1D and E).

Serologic testing was positive for antinuclear antibodies, anti-double-stranded DNA antibodies, lupus anticoagulant, and anti-cardiolipin β2 glycoprotein I antibodies. Additional findings included pericardial effusion, hypocomplementemia, proteinuria, hemolytic anemia (low haptoglobin, positive direct Coombs test) (Table 1), and erythema of the right upper arm, right palm, and anterior chest. No pulmonary or gastrointestinal lesions were observed. She was diagnosed with SLE. Treatment with glucocorticoids and cyclophosphamide was initiated, leading to rapid radiological improvement of the hepatic artery aneurysms; regression was noted on day 14 of treatment, and follow-up CT at 6 months showed near-complete resolution but also to significant clinical and laboratory improvement. Anti-double-stranded DNA antibody levels decreased, proteinuria was reduced, pericardial effusion disappeared, and anemia and hemolytic findings were ameliorated.

**Table 1.** Hematologic, Biochemical, and Immunological Findings Obtained after Hepatic Artery Aneurysm Rupture and at 6-Month Follow-Up.

Parameter	Reference range	After rupture (Day 10)	6 months follow-up
White blood cell count (/μL)	3,300-8,600	10,520	6,510
Hemoglobin (g/dL)	11.6-14.8	8.0	10.0
Platelet count (/μL)	158,000-348,000	292,000	398,000
Lactate dehydrogenase (U/L)	124-222	151	195
Haptoglobin (mg/dL)	20-200	<1	127
Direct Coombs test	Negative	Positive	NA
Serum creatinine (mg/dL)	0.6-1.1	0.75	0.45
Estimated glomerular filtration rate (mL/min/1.73 m^2^)	>60	57.1	99.5
Proteinuria (g/g creatinine)	<0.15	0.79	0.31
Aspartate aminotransferase (U/L)	13-30	46	11
Alanine aminotransferase (U/L)	7-23	26	4
Alkaline phosphatase (U/L)	38-113	122	62
Gamma-glutamyl transpeptidase (U/L)	9-32	82	29
Total bilirubin (mg/dL)	0.4-1.5	0.34	0.32
Complement component 3 (mg/dL)	86-160	67	126
Complement component 4 (mg/dL)	17-45	10	17
Total hemolytic complement activity (CH50/mL)	25-48	13.1	22.9
Anti-double-stranded DNA antibody	Negative	Positive	Negative
Lupus anticoagulant	Negative	Positive	NA
Anti-cardiolipin β2 glycoprotein I antibody	Negative	Positive	NA
Proteinase 3-anti-neutrophil cytoplasmic antibody (U/mL)	<3.4	<1	NA
Myeloperoxidase-anti-neutrophil cytoplasmic antibody (U/mL)	<3.5	<1	NA
Antinuclear antibody, Homogeneous pattern (titer)	<1:40	80	NA
Antinuclear antibody, Speckled pattern (titer)	<1:40	80	NA
Antinuclear antibody, Nucleolar pattern (titer)	<1:40	80	NA
C-reactive protein (mg/dL)	<0.14	5.01	1.48

NA: not available.

## Discussion

Etiologies of hepatic artery aneurysms include infection, atherosclerosis, fibromuscular dysplasia, vasculitis, polyarteritis nodosa, and SLE ^(3), (4)^. In autoimmune diseases, aneurysm formation is thought to result from vasculitis-induced medial layer destruction and immune cell infiltration ^(5)^. In this case, the pancytopenia 3 years earlier suggests that undiagnosed SLE had been present. The absence of positive blood cultures and of vegetations indicating infective endocarditis reduced the likelihood of an infectious aneurysm. Furthermore, regression of the aneurysms after immunosuppressive therapy supported an inflammatory etiology.

Endovascular treatment, such as TAE or coil embolization, is minimally invasive and offers benefits including shorter hospitalization and fewer postoperative complications. Open surgery, although more invasive, may provide definitive and durable treatment in cases of complex or large aneurysms ^(6)^. Hepatic artery aneurysms carry a clear rupture risk (approximately 14%), and rupture is associated with high mortality ^(3)^. Therefore, prompt diagnosis and appropriate therapeutic intervention are essential for improving prognosis. In this patient, rupture occurred during hospitalization, but timely endovascular intervention successfully achieved hemostasis and a favorable outcome.

While prior cases presented mainly with gastrointestinal bleeding or hemobilia, our patient developed acute abdominal pain from aneurysmal rupture during hospitalization ^(4), (7), (8)^. Endovascular embolization was the initial treatment in most cases, including ours, and all patients had favorable outcomes with timely intervention (Table 2).

**Table 2.** Reported Cases of Hepatic Artery Aneurysm Associated with Systemic Lupus Erythematosus.

Author & Year	Age / Sex	Chief Complaint / Presentation	Aneurysm Location	Treatment Method	Outcome	Reference
C Liu, 2011	31-year-old male	Epigastric pain and jaundice for 2 months	Two hepatic artery aneurysms in the left lateral lobe	TAE, later left lateral lobectomy and ligation of the proximal hepatic artery	Good recovery	7
EN Pollono, 2009	56-year-old female	Gastrointestinal bleeding without identifiable lesions	Multiple hepatic artery aneurysms	TAE	Good recovery	4
J Trambert, 1989	49-year-old male	Caused by ruptured aneurysms of a branch of the left hepatic artery	Branch of the left hepatic artery	TAE	Good recovery	8
Present case, 2022	74-year-old female	Abdominal pain, intraperitoneal bleeding	Multiple intrahepatic aneurysms	TAE	Good recovery

TAE: transcatheter arterial embolization.

This case highlights that rapid diagnosis, prompt hemostatic intervention, and early initiation of immunosuppressive therapy are critical for achieving favorable outcomes in SLE-associated hepatic artery aneurysms.

## Article Information

### Author Contributions

Saori Inoue: patient care, manuscript drafting. Mari Tatsumi, Yu Tanaka: clinical data interpretation, and literature review. Aya Ide: image preparation and editing.

### Conflicts of Interest

None.

### Ethics Approval and Consent to Participate

This case report did not require approval by the institutional review board, in accordance with the policy of Meitetsu hospital. Written informed consent for publication was obtained from the patient .

### Acknowledgements

None.
